# The influence of DNA repair on neurological degeneration, cachexia, skin cancer and internal neoplasms: autopsy report of four xeroderma pigmentosum patients (XP-A, XP-C and XP-D)

**DOI:** 10.1186/2051-5960-1-4

**Published:** 2013-05-08

**Authors:** Jin-Ping Lai, Yen-Chun Liu, Meghna Alimchandani, Qingyan Liu, Phyu Phyu Aung, Kant Matsuda, Chyi-Chia R Lee, Maria Tsokos, Stephen Hewitt, Elisabeth J Rushing, Deborah Tamura, David L Levens, John J DiGiovanna, Howard A Fine, Nicholas Patronas, Sikandar G Khan, David E Kleiner, J Carl Oberholtzer, Martha M Quezado, Kenneth H Kraemer

**Affiliations:** 1 Laboratory of Pathology; Center for Cancer Research, National Cancer Institute, National Institutes of Health, Bethesda, MD, 20892, USA; 2 Institute of Neuropathology, University of Zurich, Zurich, Switzerland, USA; 3 DNA Repair Section, Dermatology Branch, Center for Cancer Research, National Cancer Institute, National Institutes of Health, Bethesda, MD, 20892, USA; 4 Neuro-Oncology Branch, Center for Cancer Research, National Cancer Institute, National Institutes of Health, Bethesda, MD, 20892, USA; 5 Radiology and Imaging Sciences, Clinical Center National Institutes of Health, Bethesda, MD, 20892, USA

**Keywords:** DNA damage, DNA repair, Neurodegeneration, Glioblastoma, Cachexia

## Abstract

**Background:**

To investigate the association of DNA nucleotide excision repair (NER) defects with neurological degeneration, cachexia and cancer, we performed autopsies on 4 adult xeroderma pigmentosum (XP) patients with different clinical features and defects in NER complementation groups XP-A, XP-C or XP-D.

**Results:**

The XP-A (XP12BE) and XP-D (XP18BE) patients exhibited progressive neurological deterioration with sensorineural hearing loss. The clinical spectrum encompassed severe cachexia in the XP-A (XP12BE) patient, numerous skin cancers in the XP-A and two XP-C (XP24BE and XP1BE) patients and only few skin cancers in the XP-D patient. Two XP-C patients developed internal neoplasms including glioblastoma in XP24BE and uterine adenocarcinoma in XP1BE. At autopsy, the brains of the 44 yr XP-A and the 45 yr XP-D patients were profoundly atrophic and characterized microscopically by diffuse neuronal loss, myelin pallor and gliosis. Unlike the XP-A patient, the XP-D patient had a thickened calvarium, and the brain showed vacuolization of the neuropil in the cerebrum, cerebellum and brainstem, and patchy Purkinje cell loss. Axonal neuropathy and chronic denervation atrophy of the skeletal muscles were observed in the XP-A patient, but not in the XP-D patient.

**Conclusions:**

These clinical manifestations and autopsy findings indicate advanced involvement of the central and peripheral nervous system. Despite similar defects in DNA repair, different clinicopathological phenotypes are seen in the four cases, and therefore distinct patterns of neurodegeneration characterize XP-D, XP-A and XP-C patients.

## Background

Xeroderma pigmentosum (XP) is a rare, inherited disorder of DNA repair characterized by sun sensitivity of exposed tissues (skin, eye, oral mucous membranes) and a greater than 10,000-fold increased risk of cutaneous, ocular, and tongue neoplasms [1-4]. Some XP patients also have neurologic manifestations [3-11]. There are seven DNA nucleotide excision repair (NER)-deficient complementation groups designated XP-A through XP-G and an XP variant form with defective trans-lesion DNA polymerase eta [12,13]. Recently we reported on 106 XP patients seen at NIH over the past 4 decades [1]. Progressive neurologic degeneration was present in 24% (25/106), primarily in XP-D (n=16) and XP-A (n=6) complementation groups. Their core clinical features included loss of intellectual function, abnormal speech, mutism, areflexia, ataxia, peripheral neuropathy, and loss of the ability to walk. Progressive sensorineural hearing loss was a strong indicator of neurological degeneration [14]. The postmortem findings of XP, including involvement of the nervous system, have been described in the literature [6,7,10,15-34]. Most of these patients were in complementation group XP-A. There were no post-mortem reports on XP-D patients.

**Figure 1:**
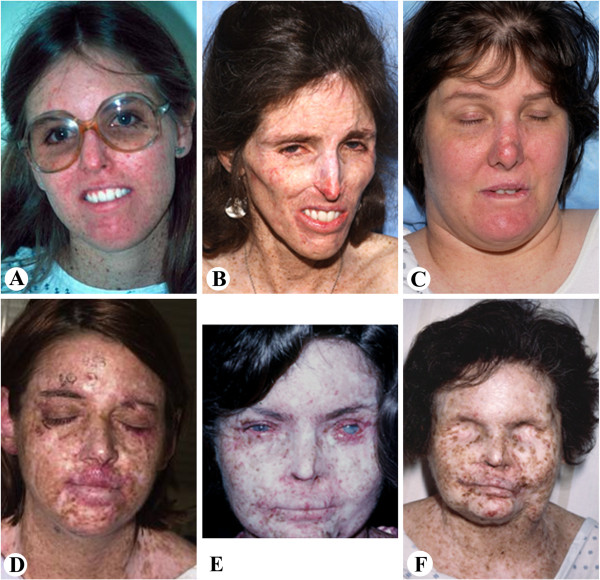
**XP patients studied. A** and **B**: Case 1 XP-A patient (**A**) at age 17y with numerous freckle-like pigmented lesions on sun exposed skin and (**B**) at 37y with marked cachexia and thinning of subcutaneous tissues of face and chest. She had more that 100 surgical procedures on her face for removal of skin lesions. **C**: Case 2 XP-D patient at age 40y. She had been well protected from sun exposure since early childhood and had only few pigmented lesions and skin cancers. **D**: Case 3 XP-C patient, at age 29y with multiple freckle-like pigmented lesion on sun exposed skin and cheilitis. The patient underwent many surgical procedures for removal of skin cancers on her face. **E** and **F**: Case 4 XP-C patient, at age 28y (**E**) with multiple pigmented lesions, telangiectasia, cheilitis and corneal clouding. Multiple surgical procedures were performed on her face for removal of skin cancers and at age 48y (**F**) following exenteration of both orbits for treatment of ocular squamous cell carcinomas.

Here we report the post-mortem findings in 4 XP cases with different clinical features (Figure 1 and Table 1). The XP-A and XP-D patients showed severe progressive neurologic deterioration. The XP-A and XP-C patients presented with hundreds of skin cancers. The two XP-C patients died of internal neoplasms including glioblastoma in XP24BE and cervical adenocarcinoma with tumor metastasis to multiple organs and systems in XP1BE.

**Table 1:** **Clinical and autopsy abnormalities in XP**-**A** (**XP12BE**) **and XP**-**D** (**XP18BE**) **patients compared to two XP**-**C patients**

	**Case 1**	**Case 2**	**Case 3**	**Case 4**
	XP-A (XP12BE) (GM05509)*	XP-D (XP18BE) (CRL1275)**(XPKABE)	XP-C (XP24BE) (GM11638)*	XP-C (XP1BE) (GM10881)*
	late onset juvenile form XP	XP with neurological degeneration	no neurological disease	subclinical neurodegeneration
Age at death/Gender/DOB	d44 yr/F b1965	d45 yr/F b1964	d35 yr/F b1972	d49 yr/F b1944
XP complementation group	XP-A	XP-D	XP-C	XP-C
Mutations (see text for details)	compound heterozygote	compound heterozygote	compound heterozygote	homozygous
**CLINICAL FINDINGS**				
Height	145.5 cm (<3 %ile)	165 cm (50–75 %ile)	153.3 cm - 34 yr (10 %ile)	160 cm (25–50 %ile)
Weight	32 kg (<3 %ile)	60.6 kg (50 %ile)	56.7 kg - 35 yr (50 %ile)	76.6 kg (50–75 %ile)
Cachexia?	yes	no	no	no
Acute skin sunburning on minimal exposure?	yes	yes	no	no
Freckle-like skin lesions on sun exposed skin?	yes	yes	yes	yes
Skin cancer?^1^	>200 BCC and 1 SCC	7 SCC and 2 BCC	>200 (190-BCC,3 SCC and ~50 MIS)	>200 (>35 BCC, >37 SCC, 2 soft tissue sarcoma, 28 MIS and 6 Mel)
Microcephaly?	yes (2%ile)	no	no	no
Hearing	progressive high frequency sensorineural hearing loss	progressive high frequency sensorineural hearing loss	normal	subclinical high frequency sensorineural hearing loss at 48 yr
Deep tendon reflexes	absent	absent (1995)	normal	normal at 37 yr, ankle hyporeflexia at 43 yr
Able to walk?	no	no	yes	yes
Able to talk?	no	no	yes	yes
Able to care for self?	no	no	yes	yes
Difficulty swallowing?	yes - G-tube - age 37 yr	yes - G-tube - age 44 yr	normal	normal
Primary ovarian failure?	no	unknown	yes	yes
Anterior eye abnormalities	bilateral pinguecula, exposure keratopathy^2^	bilateral pinguecula, exposure keratopathy^2^	corneal scar, pterygium	blateral orbital exenterations for infiltrative SCC of globe3
Eye - retinal degeneration	optic nerve atrophy^2^	no^2^	no	unknown
Imaging of brain	diffuse cerebral and cerebellar atrophy (41 yr −2006)	minimal cortical atrophy (19 yr- 1983)	left frontal lobe tumor	slight cerebral cortical atrophy and ventricular enlargement (44 yr)
**AUTOPSY FINDINGS**				
Thick calvarium?	no	yes; cortical sclerosis, no tumor seen	no	no
Brain weight [normal 1240 g (1050–1550)]	660g (~ 6mo)	760g (~1 yr)	1330g [normal]	1300g [normal]
Brain atrophy?	yes - diffuse	yes - diffuse	no atrophy - tumor	no, except optic nerves secondary to orbital exenteration
Dilated brain ventricles?	yes	yes	asymmetric due to tumor	no
Thin corpus callusum?	yes	yes	no remarkable features	no
Histological neuronal loss?	yes - hippocampus, pons, medulla, midbrain, thinned cortex, small cerebellum	yes – outer cortex (neuronal loss and vacuolization resembling status spongiosis), hippocampus (CA2 and CA3 regions), basal ganglia, cerebellum	no remarkable features	no
Histological gliosis?	yes - midbrain, pons, medulla, basal ganglia, thalamus, hippocampus	yes - cortex, hippocampus	no remarkable features	yes - molecular layer of cerebellum
Histological myelin pallor?	yes - temporal lobe, frontal lobe, cerebellum	yes - basal ganglia, cerebellum	no remarkable features	no remarkable features
Cerebellum	Atrophy, loss of Purkinje cells with axonal swelling, Bergmann astrocytosis	atrophy, patchy Purkinje cell loss	no remarkable features	moderate to marked Purkinje cell loss with Bergmann astrocyte proliferation
Dorsal root ganglia	no remarkable features	no remarkable features	no remarkable features	severe neuronal loss
Histology of peripheral nerves	minimal focal perivascular inflammation in the adjacent connective tissue	no pathologic changes	femoral nerve - unremarkable	median nerve mild focal interstital fibrosis, sural nerve - no pathologic diagnosis
EM of peripheral nerves	axonal neuropathy	not done	not done	not done
Eye pathology?	neovascularization of cornea, optic atrophy^2^	neovascularization of cornea, cataract^2^	not done	sockets of orbits lined with skin
Histology of muscles	myofiber type -grouping	no pathologic changes	unremarkable	angulated fibers of skeletal muscles
Histology of Pharynx	inflammation and fibrosis	chronic inflammation	normal	normal
Esophagus	no pathologic changes	T-lymphocyte infiltration of Auerbach's plexus	no pathologic changes	no pathologic changes
Larynx	no pathologic changes	ulceration with chronic and acute inflammation	pink mucosa	delicate pink mucosa
Lungs	normal	bronchopneumonia	bilateral pneumonia	pulmonary emboli
Thyroid	normal	normal	cystic nodule filled with pink, amprphous material, consistent with goiter	follicular adenomas
Ovaries	no pathologic changes	no pathologic changes	small - microscopic fibrosis, no follicles	covered by tumor
Uterus	leiomyomas	adenomyosis	small - calcified nodules 1 cm, leiomyomas	covered by tumor
Breasts	fibrocystic changes	fibrocystic changes	no masses	no masses
Cause of death	XP-related neurologic degeneration	XP-related neurologic degeneration	Glioblastoma WHO grade IV. Tumor cells positive for GFAP and IDH1, negative for EGFR. P53 positive in <5% tumor cells.	metastasis of well differentiated mucinous adenocarcinoma of uterine endocervix

^1^BCC - basal cell carcinoma, SCC -squamous cell carcinoma, MIS - melanoma in situ ^2^From Ramkumar et al. (reference 26) ^3^From Gaasterland et al. (reference 48) *Coriell Institute for Medical Research cell culture identification number **American Type Culture Collection cell culture identification number.

## Results

### Case histories

#### Case 1 (XP12BE - XP-A)

The patient was a 44 year-old female with XP who had been followed at NIH since diagnosis at age 4 years [4,8,9,14,26,35] (Figure 1A and B and Table 1). She experienced the first acute sun-sensitivity reaction at age 3 months with erythema and swelling after minimal sun exposure. Over time she developed freckle-like pigmentation on the sun exposed skin of her face, neck and dorsal upper extremities. Her first pathologically documented skin malignancy was a sclerosing basal cell carcinoma of the cheek at age 8 years. Subsequently, the patient developed more than two hundred skin cancers, which were mostly basal cell carcinomas (BCC) with 1 squamous cell carcinoma (SCC). At age 19 years, she was treated with oral 13-cis retinoic acid for skin cancer prevention [36]. She responded well with reduction in new skin cancer frequency from 43 BCC in two years before treatment to 3 new BCC after two years of treatment. Eighteen months after the discontinuation of 13-cis retinoic acid, she developed 18 new skin cancers. In the following years, she practiced increased protection from the sun with improved protective clothing and sunblocking agents. She experienced progressive neurologic decline, which further contributed to reduced outdoor exposure. Over time, fewer skin cancers developed and her overall XP-related skin pigmentation faded with only few lesions present.

**Figure 2:**
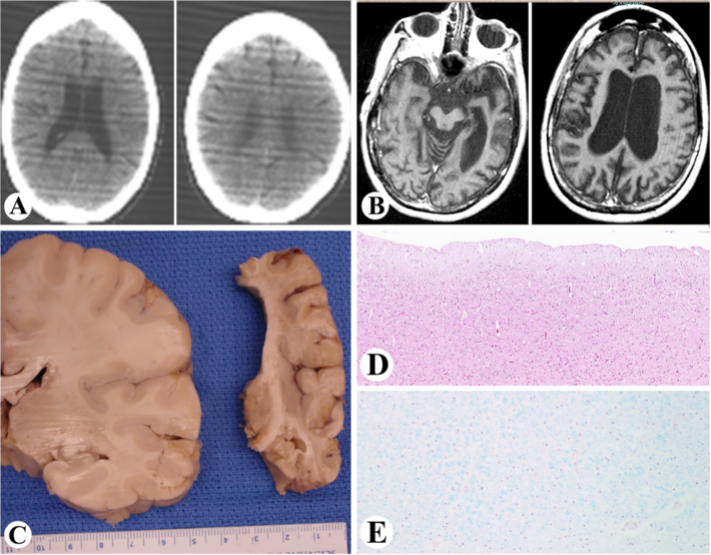
**Case 1.** XP-A **A**: Two consecutive Computed Tomography (CT) images of the brain obtained at age 20y. There are no focal brain abnormalities and no appreciable brain atrophy. **B**: Two axial T1-weighted Magnetic Resonance Images (MRI) of the brain in the same patient obtained at age 41y. There is evidence of prominent brain atrophy manifested by enlargement of the ventricles and widening of the intracranial subarachnoid spaces. **C**: Gross atrophy of the right brain hemisphere (right, comparable image to 2**B**) in comparison to the normal brain at the same age and gender (left). Loss of brain substance appears to be greater in the white matter. **D**: Thinning of cortex of the right temporal lobe (H&E, x 40). **E**: Luxol fast blue stain of the right temporal lobe showing patchy loss of myelination.

While her early developmental milestones were within normal range, neurological signs were noted at age 7 years, with loss of deep tendon reflexes. At age 8 years, an audiogram revealed bilateral hearing loss at 8,000 Hertz, which progressed over the years [9,14]. Learning difficulties and an IQ of 79 were noted at age 15 yr. Computerized tomography (CT) of the brain at age 20 showed no focal abnormalities and no appreciable atrophy (Figure 2A). At age 21 years, truncal ataxia became more prominent and tongue movement appeared uncoordinated. At the age of 22, she began to lose pain sensation, the Romberg test became positive and she exhibited child-like behavior [9]. By age 37 years, she was immobile and required assistance to walk. Because she was unable to swallow solid food without choking a gastrostomy tube (G-tube) was placed for nutritional support. Despite this therapeutic measure, she continued to become markedly cachectic. At age 41 years, there was evidence of prominent brain atrophy on imaging studies (Figure 2B). Neurological deterioration progressed inexorably and she became completely unresponsive and expired at the age of 44. An unrestricted autopsy was performed. Compound heterozygous mutations were detected in the *XPA* DNA repair gene (Table 1) with G>T intron 3 splice acceptor, and G>C splice donor of exon 4 [37,38]. The post-UV DNA repair rate (unscheduled DNA synthesis (UDS)) of the cultured skin fibroblasts was about 1% of normal [4,35,39,40]. Post-UV survival of cultured skin fibroblasts was markedly reduced [41].

### Case 2 (XP18BE- XP-D)

This 45-year-old female patient with XP had a history of progressive neurologic deficits [14,26] (Figure 1C and Table 1). She presented to the NIH at age 5 years with hearing difficulty and developmental delay. In addition, she had marked photosensitivity with blistering on minimal sun exposure. At age 8 years, SCC was removed from her nose. Subsequently, she practiced extensive early sun protection and only developed 9 skin cancers during her lifetime (2 BCC and 7 SCC). At age 13 years, she was first noted to have high frequency sensorineural hearing loss and at age 30 years, a repeat audiologic evaluation revealed profound sensorineural hearing loss [14]. At age 31 she developed ataxia with a broad-based gait, eventually requiring confinement to a wheelchair at age 36. By age 39 years, she was unable to care for herself and needed to read lips and write notes in order to communicate. She developed progressive dysphagia and at age 44 years a gastrostomy tube was placed for nutritional support that proved successful for weight maintenance. Her final admission was for pneumonia and sepsis. She expired 3 days later and an unrestricted autopsy was performed. Laboratory testing demonstrated XP-D complementation group (Table 1) with compound heterozygous mutations in the *XPD* (*ERCC2*) gene: R616P and R683W. Cultured skin fibroblasts (XPKABE) showed post-UV UDS of 25-50% of normal but markedly reduced post-UV survival [41].

### Case 3 (XP24BE- XP-C)

**Figure 3:**
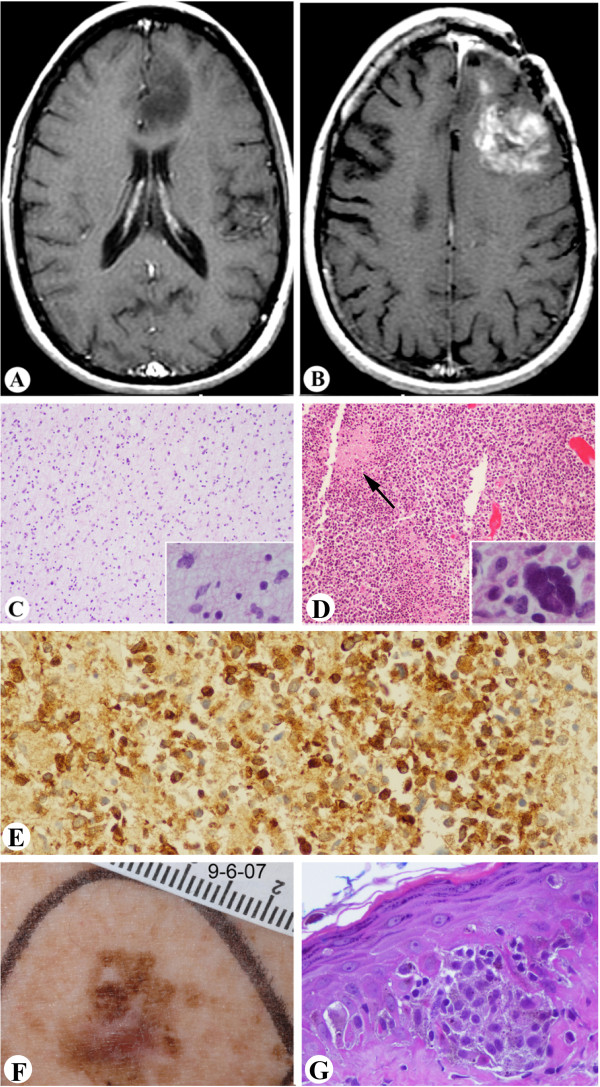
**Case 3.** XP-C **A**-**B**: MRI images of brain tumor. **A**: Axial post contrast T1-weighted MRI image of the brain obtained at age 29y showing a space occupying lesion in the left frontal lobe. The lesion demonstrates decrease signal intensity and does not enhance. The lack of enhancement after contrast indicates low histologic grade of this tumor. **B**: Axial post contrast T1-weighted MRI image of the brain in the same patient obtained five years later. There is evidence of tumor recurrence after surgery. The tumor demonstrates abnormal increased enhancement on the post contrast scan. This feature is indicative of malignant transformation. **C**: Histologic features of the primary tumor (as shown in **A**), a low grade astrocytoma (inset shows slightly atypical cells). **D**: Histologic features of the recurrent tumor, a glioblastoma. The tumor is characterized by increased cellularity, and pseudo-palisading necrosis (arrow), multinucleated tumor cell is shown in inset. **E**: Immunohistochemistry shows that the tumor cells are positive for IDH1 (X200). **F**: Pigmented lesion showing irregular size, shape and color. **G**: Histology of F showing melanoma in situ (x400).

Case 3 was a 35-year-old female diagnosed with XP at age 6 years (Figures 1D and 3 and Table 1). During her disease course, she developed almost 200 skin cancers including BCC, SCC, 11 melanomas in situ (Figure 3G and H), and one invasive melanoma (0.65 mm). At age 11 years, she participated in a study with oral 13-cis retinoic acid (isotretinoin) for skin cancer chemoprevention with a good response [36]. She developed premature menopause before age 30 years.

At age 29 years, she was found to have an asymptomatic left frontal lobe, non-enhancing brain mass lesion (Figure 3A). The patient elected follow-up with serial MRIs. However, two years later, the lesion enlarged and became symptomatic. She underwent craniotomy with subtotal resection of the mass. Pathologic examination revealed a low grade astrocytoma (Figure 3C and D). She was treated postoperatively with external beam radiation therapy with a total dose of 5,940 cGy. As with other XP patients receiving radiotherapy [42], she had a normal skin response. The patient was stable until age of 34 years, when she developed expressive aphasia and fatigue. Imaging revealed recurrent tumor with abnormal increase of enhancement on the post-contrast scan (Figure 3B), a feature considered indicative of malignant transformation. Resection showed that the tumor had progressed to glioblastoma (Figure 3E and F). Worsening aphasia and right-sided hemiparesis complicated her postoperative course. She began temozolomide therapy. She did not have any unusual side effects to standard doses of temozolomide which was well tolerated. The patient’s condition continued to deteriorate, and she expired at home. A partially restricted autopsy was performed. Laboratory testing showed compound heterozygous mutations in the *XPC* gene (Table 1): A155X and 83bp insertion of intron 5.1 with stop codon 34 codons downstream [43].

### Case 4 (XP1BE- XP-C)

Case 4 was a 49-year-old Caucasian female with XP, who had been followed at the NIH since age 24 years [4,10,14,44,45] (Figure 1E and F and Table 1). She had more than 200 biopsy-proven BCC and SCC and developed approximately 38 separate primary melanomas, half of which were in-situ lesions [46]. A number of these lesions responded to intra-lesional alpha interferon [47]. Bilateral orbital exenterations were performed for recurrent squamous cell carcinomas of the cornea and globe at age 32 and 36 years, followed by 5,000 rads of irradiation with a normal response [48]. In addition, she was found to have subclinical, asymptomatic neurologic disease, including right peroneal nerve weakness and subclinical hearing loss, without mental deterioration [10,14,44]. She had multiple urinary tract infections, chronic glomerulonephritis following left nephrectomy at age 40 years for staghorn calculi, premature menopause secondary to hypogonadotropic hypogonadism, and fatty liver. Just prior to death, she experienced shortness of breath and developed a superaventricular tachycardia. An unrestricted autopsy was performed [10]. She had mutations in the *XPC* gene (Table 1): homozygous deletion of AA Exon 8 del AA 1396–7, fs188 - stop 5 codons downstream [49]. Cultured skin fibroblasts had about 10% of normal post-UV UDS [4,35,39]. Post-UV skin fibroblast cell survival was reduced [41].

## Pathological examinations

### Case 1 (XP12BE- XP-A)

#### Neuropathology

Gross description: The brain weighed 660 g (normal for ~ 6 month old infant) and was bisected fresh (Figure 2C and Table 1). The leptomeninges were smooth and transparent. There was no venous congestion or evidence of acute subarachnoid hemorrhage. Generalized brain atrophy with marked dilatation of lateral ventricles and thinning of the corpus callosum was noted. The gyri/sulci were grossly within normal limits. Examination of the basal cerebral vasculature revealed no dilatation, thrombosis, atheroma, or other abnormalities. Coronal sectioning of the right cerebral hemispheres confirmed diffuse atrophy (Figure 2C) with marked ventricular dilatation. While the cortical ribbon was thin throughout the hemispheres, the loss of brain substance seemed greater in the subjacent white matter. Deep gray structures including the mamillary body, thalami, subthalamic nuclei, basal ganglia, and the hippocampus were grossly unremarkable. Serial sectioning of the right brainstem and cerebellum revealed no grossly significant abnormalities. The spinal leptomeninges were thin and translucent and the subarachnoid space was free of exudates. The spinal arteries and veins were unremarkable. Significant eye findings included exposure keratopathy and bilateral optic atrophy as previously described [26].

**Figure 4:**
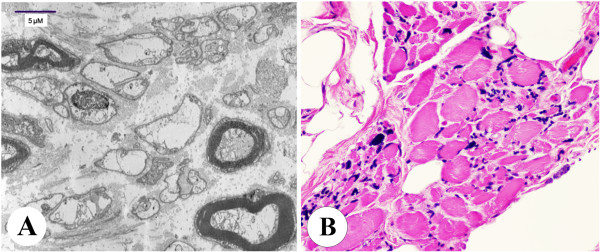
**Case 1.** XP-A Axonal neuropathy and denervation atrophy of skeletal muscle. **A**: Electron microscopy showing mild to moderate reduction in the number of large myelinated axons accompanied by increased collagen and axonal degeneration and axonal atrophy consistent with axonal neuropathy. **B**: Skeletal muscle showing marked variation in fiber size, with rounded and angulated atrophic fibers, pyknotic nuclear clumps, fibrosis with fat replacement.

Microscopic description of sections from the midbrain, pons, medulla and hippocampus revealed neuronal loss and gliosis. In addition increased capillary density was seen in the thalamus. Sections from the right frontal and temporal cortex (Figure 2D) showed neuronal loss and gliosis accompanied by microglial activation. In addition, patchy white matter rarefaction was seen and highlighted by a LFB stain (Figure 2E). There were no neuritic plaques, tangles or neuronal inclusions. Sections from the right cerebellum demonstrated overall atrophy of the folia. Focal loss of Purkinje cells with increased numbers of torpedoes (Purkinje cell axonal swellings) and associated Bergmann astrocytosis was seen at the junction of the granular and molecular layers. The granular layer was mostly preserved with only mild rarefaction. A section from the dura was unremarkable. Electron microscopy of the radial nerve showed increased interstitial collagen with reduced density of large and myelinated fibers, axonal atrophy and occasional regeneration clusters consistent with axonal neuropathy (Figure 4A). Rare thinly myelinated fibers were seen. Denervation atrophy (esterase positive angular atrophic fibers, pyknotic nuclear clumps) of the skeletal muscles was present. Fiber type grouping, however, was not a feature. Temporal bone histology revealed marked atrophy of the cochlear sensory epithelium and neurons [14].

Other findings: The indicated cause of death was XP-related neurologic degeneration. Hemangioma of the lip, seborrheic keratosis, uterine leiomyoma and fibrocystic change in bilateral breasts were also identified (Table 1).

### Case 2 (XP18BE- XP-D)

#### Neuropathology

**Figure 5:**
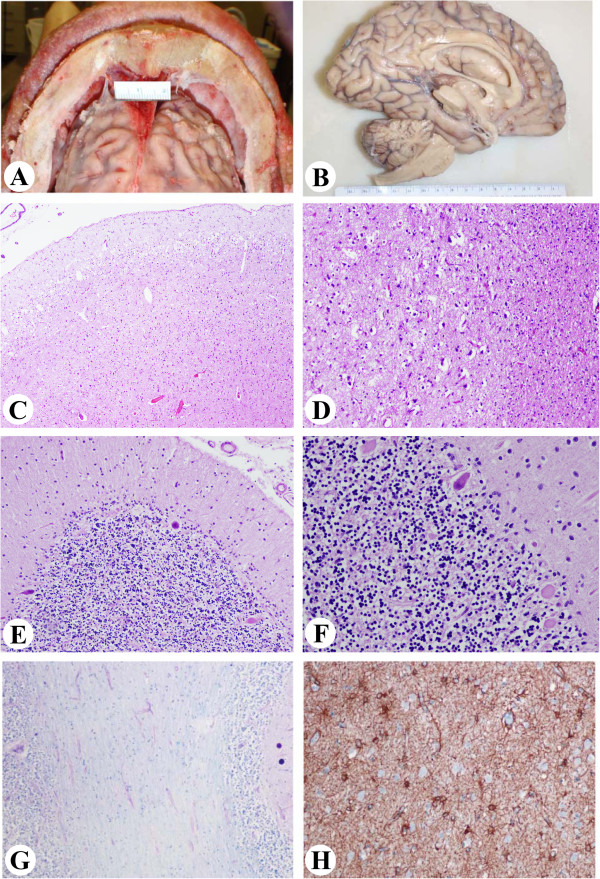
**Case 2 XP-D Neuropathologic changes. A**: Thick calvarium (2.3 cm, frontal). **B**: Global cerebral atrophy with dilated ventricles and thinned corpus callosum. **C**-**D**: Thinning of the cortical ribbon with vacuolization of the neuropil, neuronal loss and gliosis in the right frontal (**C**, x100) and parietal cortex (**D**, x200). **E-F:** Patchy loss of Purkinje cells in the right cerebellum with Bergman gliosis and axonal torpedos (**E**, x40; **F**, x200). **G**: Patchy myelin pallor in the right cerebellum (x100). **H**: GFAP highlights the reactive astrocytes in parietal lobe (x200).

Gross description of the skull was notable for a substantially thickened calvarium, measuring 2.3 cm (frontal) (Figure 5A), 1.7 cm (occipital) and 1.0 cm (temporal). There were two boney excrescences measuring 1.4x1.2x0.4 cm and 1.2x1.0x0.6 cm in the right middle fossa of the skull base. The brain weighed 760 g (normal for ~ 1 yr old child) and showed diffuse widening of the sulci consistent with global atrophy (Table 1). The cerebral gyri/sulci were unremarkable and the hemispheres were symmetric. Coronal sections of the hemispheres confirmed the diffuse cerebral atrophy with dilated ventricles and a thin corpus callosum, but no focal lesions were seen (Figure 5B). Cerebellar hemispheres and vermis also appeared atrophic. Examination of peripheral nerves including both brachial plexuses, and radial, median, ulnar, femoral, sural and vagal nerves was unremarkable.

**Figure 6:**
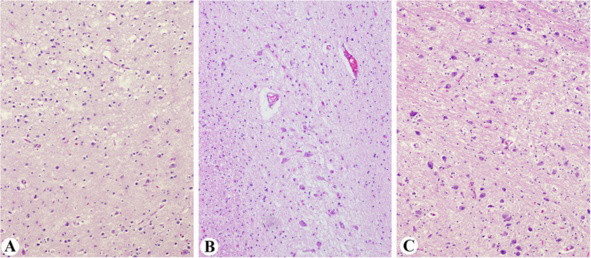
**Case 2 XP-D.** Hippocampal sections showed neuronal loss involving CA2/CA3 sectors (**A**, X200); Neuropil disorganization seen in basal ganglia (**B**, X200) and midbrain-pontine areas (**C**, X200).

Microscopic description: Sections from the right frontal and parietal cortex showed thinning of the cortical ribbon with extensive neuronal loss widespread vacuolization of the neuropil (Figure 5C and 5D). Vacuolization was most prominent in the deep cortical layers (resembling the pattern of coarse vacuolation termed status spongiosis); neuronal loss and gliosis were accompanying features. Neurofibrillary tangles, plaques or other inclusions or storage products were not identified. Sections from the hippocampus demonstrated atrophy, gliosis, and pyramidal cell loss and vacuolization of the neuropil. Hippocampal neuronal loss most prominently involved the CA2 and CA3 sectors. Sections from the right basal ganglia and mid brain-pontine junction revealed extensive neuronal loss and gliosis, Figure 6 (A-C). Sections from the right cerebellum showed focal loss of Purkinje cells and accompanying Bergmann gliosis (Figure 5E and 5F) with patchy myelin pallor of the cerebellar white matter (Figure 5G). A section from the grossly described boney excrescence in the right middle fossa showed cortical sclerosis with no evidence of tumor. Immunohistochemistry and special stains for GFAP and LFB were performed on sections from the cortex, hippocampus and cerebellum. The GFAP stain highlighted reactive astrocytes (Figure 5H).

**Figure 7:**
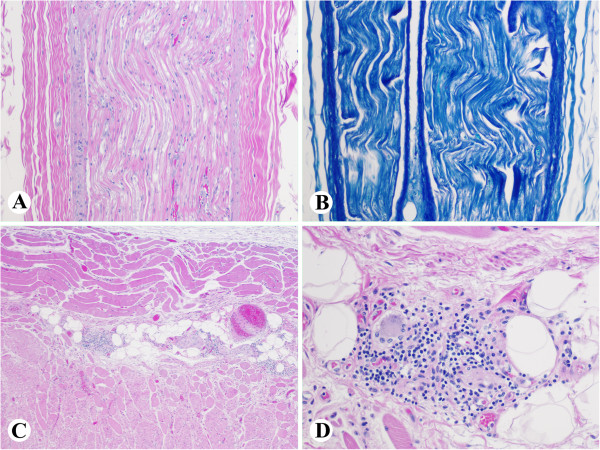
**Case 2 XP-D.** Neuropathologic changes. **A** and **B**, No specific abnormality was appreciated in peripheral nerve stained with H&E (**A**, x100) and LFB/PAS (**B**, x100). **C** and **D**, Periganglial lymphocytic infiltration of Auerbach’s plexus in the esophagus (**C**, x40; **D**, x 200).

Histology of the peripheral nerves (Figure 7A and 7B), pharyngeal muscle and esophageal muscle were unremarkable. Examination of the esophagus showed infiltration of the ganglia of Auerbach’s plexus by CD3 positive T-lymphocytes (Figure 7C and 7D).

Other findings: The immediate cause of death was aspiration pneumonia. Focal patchy consolidation, bronchial mucus plugging and a cluster of plant (vegetable) cells with alveolar and interstitial infiltration by neutrophils and lymphocytes were seen in both lungs. A small (0.2 cm), hemangioma was present on the patient’s back. Laryngeal ulceration, bilateral breast fibrocystic changes, mild renal glomerulosclerosis, subserosal uterine adenomyosis, and cholelithisasis were also present (Table 1). There was no ocular neoplasm identified. The eye findings were reported previously [26], and consisted of bilateral pinguecula, corneal pannus, exposure keratopathy, retinal gliosis and bilateral peripheral retinal pigmentary degeneration.

### Case 3 (XP24BE- XP-C)

#### Neuropathology

Gross findings: There were several metal plates on the frontal bone indicating prior surgery. The cranial vault appeared of normal thickness. The brain weighed 1330 g fresh (normal for adult) (Table 1). The leptomeninges over the left frontal region were slightly distorted with multiple sutures; the leptomeninges over the right hemisphere were unremarkable. The entire brain appeared edematous and the left cerebral hemisphere was enlarged, and with midline shift, but no obvious herniations were identified. Examination of the arteries of the circle of Willis and their major branches revealed no evidence of atherosclerosis, thrombus formation or malformations. The superficial veins and cranial nerves were unremarkable. Coronal sectioning of the cerebral hemispheres revealed an ill-defined tumor mass infiltrating the left frontal and temporal lobes, crossing the corpus callosum and invading the right cerebral hemisphere. The tumor was tan/white, soft with grey gelatinous areas, resembling tumor necrosis. Serial transverse sectioning of the brain stem and cerebellum revealed no abnormalities. The spinal cord was grossly unremarkable.

Microscopic findings: The prior primary tumor as shown in Figure 3A was a low grade astrocytoma (Figure 3C) with only slightly pleomorphic tumor cells (Figure 3C inset) that stained positive with GFAP. The proliferative index of the tumor cells was low as indicated by the MIB-1 staining. At autopsy, the diffusely infiltrative astrocytic tumor showed high- grade features characterized by frequent mitotic figures, microvascular proliferation, and pseudopalisading necrosis (Figure 3D). Scattered multinucleated tumor cells were seen (Figure 3D inset). The tumor cells immunolabeled with GFAP and IDH1 (Figure 3E), and were negative for EGFR. MIB-1 stain indicated a high proliferative rate and p53 stain showed focal positivity in less than 5% of tumor cells. The sections from the remaining brain and spinal cord did not show significant pathologic changes.

Other findings included bilateral pneumonia with bacterial and fungal infection, which was the immediate cause of her death (Table 1). The ovaries were small with microscopic fibrosis with no follicles observed. A cutaneous melanoma in situ (Figure 3F and 3G) and a basal cell carcinoma were identified a few weeks before the patient expired. Several pigmented skin lesions were sampled at autopsy, including lentiginous melanocytic proliferation, atypical melanocytic proliferation, severely atypical melanocytic proliferation and seborrheic keratosis. No other skin cancers were identified.

### Case 4 (XP1BE- XP-C)

#### Neuropathology

Gross description: The brain weighed 1300 gm (normal for an adult) (Table 1) and was bisected fresh. The dura, cerebral hemispheres, and infratentorial structures were externally unremarkable. The optic nerves and chiasm were atrophic. There was patchy non-occlusive atherosclerosis involving the basilar artery and circle of Willis. Sequential sections through the hemispheres, brain stem, and cerebellum revealed no focal lesions. The spinal arteries and veins were unremarkable. Peripheral nerves including median, sural nerves and celiac plexus revealed no gross abnormalities.

**Figure 8:**
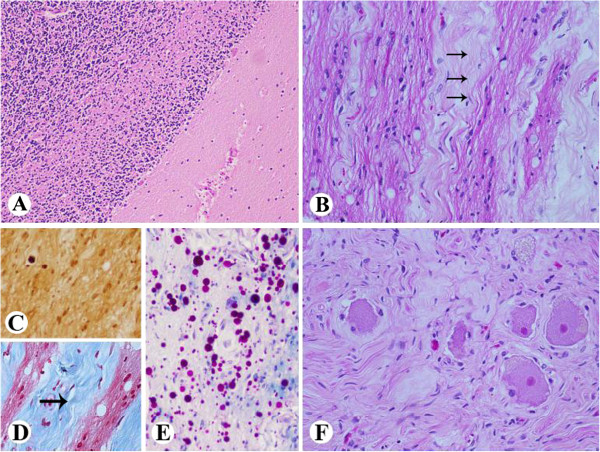
**Case 4 XP-C Neuropathologic changes. A**: The cerebellum shows focal loss of Purkinje cells (x100). **B**-**E**: Sections of optic nerves and geniculate nuclei reveal degenerative changes, including axonal loss, gliosis, and fibrosis (arrows) in keeping with the patient’s 10 year history of bilateral orbital exenterations (**B**. H&E x200; **C**. immunostain for Bielschowsky, x200 **D**. Masson stain, x200, **E**. LFB/PAS stain, x200). **F**. Dorsal root ganglia show severe neuronal loss and accompanying fibrosis (H&E, x200).

Microscopic description: The cerebellum showed focal loss of Purkinje cells (Figure 8A) with accompanying Bergmann astrocyte proliferation, and gliosis of the molecular layer. The hemispheric cortical grey matter and subcortical white matter were unremarkable. The pituitary was unremarkable. Sections of optic nerves and geniculate nuclei revealed degenerative changes (Figure 8B and 8C) including marked axonal loss with gliosis and resultant fibrosis of the fibro-vascular bundles and trans-synaptic degeneration in the lateral geniculate body with gliosis, consistent with the patient’s 10-year history of bilateral orbital exenterations [48]. Sections of spinal cord revealed no pathologic changes; however, the dorsal root ganglia showed severe neuronal loss with sclerotic foci occupying previous neuronal sites (Figure 8D) as reported previously [10]. Peripheral ganglia (celiac) were unremarkable.

**Figure 9:**
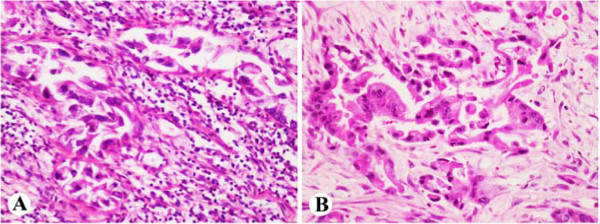
**Case 4 XP-C Carcinomatosis.** Well differentiated mucinous adenocarcinoma of endocervical origin (**A**, x200), caused extensive abdominal carcinomatosis and involved diaphragm (**B**, x200).

Multiple pulmonary emboli were the immediate cause of her death. The underlying cause of the pulmonary emboli was a clinically unsuspected well differentiated mucinous adenocarcinoma of endocervical origin (Figure 9A), which directly invaded the surrounding pelvic organs including ovaries and sigmoid colon, causing diffuse abdominal carcinomatosis that involved the diaphragm (Figure 9B), omentum, appendix, large and small bowel, and metastasized to multiple splenic hilar lymph nodes, spleen and lungs.

Other findings included obesity, glomerulonephritis, multiple simple renal parenchymal cysts, steatohepatitis, nodular regenerative hyperplasia of liver, right ventricular dilatation and left ventricular hypertrophy, and thyroid follicular adenomas. In addition, there was mild to moderate atherosclerosis of the left anterior descending artery, and moderate to severe atherosclerosis of the right coronary artery.

## Discussion

### Neurodegeneration in XP patients

The present study represents the first detailed description of the postmortem neuropathologic findings in an XP-D patient. These features have been systematically compared to an XP-A and two XP-C patients (Table 1). Of note, all four patients were included in a large series of XP patients followed at the NIH for many years permitting close observation of disease progression and the effects of treatment [1,14]. Major clinical neurologic abnormalities in XP patients include sensorineural hearing loss, peripheral neuropathy, microcephaly and dementia. The XP-A and XP-D patients were diagnosed with XP at age 4 and 5 years, respectively, and developed high frequency sensorineural hearing loss at a young age. Their neurologic status showed progressive decline with loss of ability to walk and talk leading to inability to care for themselves and death at age 44 and 45 years of age (Table 1).

The core neuropathologic findings of this study are profound global cerebral and pancerebellar atrophy in the XP-A and XP-D cases. The most striking gross finding was generalized brain atrophy (660 and 760 g) with brain size similar to that of a 6 to 12 month old infant [50]. Consistent with what has been described previously in XP-A patients [4-6,11], we found brain atrophy involving all lobes. There was diffuse neurodegeneration with neuronal loss and gliosis of both hemispheres, similar changes in the cerebellum, basal ganglia, and corticospinal tracts. Retinal gliosis was identified in both cases [26]. In addition, the XP-D patient had a thickened calvarium, vacuolization of the deep cortical neuropil, patchy Purkinje cell loss, and infiltration of the ganglia of esophageal Auerbach’s plexus by T-lymphocytes. Intriguingly, calvarial thickening which has been described in XP-A patients from Finland [5] was not observed in the XP-A patient.

Both XP-A and XP-D patients developed dysphagia, which poses difficult challenges for clinical management. In addition to CNS dysfunction, we carefully evaluated the peripheral neuromuscular system, and found axonal neuropathy and chronic denervation atrophy of skeletal muscle including those that related to swallowing in the XP-A patient. The peripheral neuropathy has been previously reported and involves both motor and sensory nerves. The underlying pathogenic mechanism is thought to be a neuronopathy [7,51]. In contrast, significant peripheral neuronopathy was not identified in the XP-D patient where section of the esophagus showed T-cell infiltrates of Auerbach’s plexus. This finding suggests an immune-mediated mechanism may be associated with acute autonomic and sensory neuropathy leading to impairment of both superficial and deep sensation in the XP-D patient.

Both the XP-A and the XP-D patients had severe progressive neurodegeneration. However, while the swallowing difficulty led to the placement of a G-tube for feeding of both patients, only the XP-A patient developed cachexia. The XP-D patient retained her weight, which suggests that cachexia is not directly related to the extent of neurodegeneration or the inability to swallow.

A patient with XP/Cockayne syndrome complex was described with a defect in the XP-G gene (XP20BE) [27]. He died at age 6 yr and had marked growth retardation with short stature and severe cachexia. However the autopsy findings of XP20BE were different from the XP-A and XP-D cases. His brain appeared proportionally small and not atrophic with a grossly normal cortical ribbon and an unusual tigroid pattern of demyelination. In addition, the patient had cataracts, and retinal pigmentary atrophy typical of Cockayne syndrome.

With respect to the two XP-C cases, one case showed unexpected carcinomatosis with endocervical adenocarcinoma. The other patient developed a glioblastoma, which arose from a pre-existing low-grade, diffuse astrocytoma. This observation is furthered supported by the IDH1 labeling of the tumor cells. It has been reported that as high as 90% of diffuse and anaplastic gliomas and secondary glioblastomas (arising from a pre-existing low-grade astrocytoma) are IDH1-positive [52]. The generalized neurodegenerative changes seen in the XP-A and XP-D cases were not observed in the XP-C cases although the older patient had focal loss of Purkinje cells and, neuronal loss in the dorsal root ganglion.

There was a clinical correlation between the skin abnormalities and the presence of progressive neurological degeneration. In a longitudinal study of 73 XP patients with audiograms examined at the NIH, all 17 patients who developed sensorineural hearing loss and progressive neurological degeneration had a history of acute burning on minimal sun exposure, including the XP-A and XP-D patients in this report [14]. This burning history was not present in the XP-C patients and they did not show progressive neurological degeneration. Further, cultured skin cells from XP patients with a history of burning on minimal sun exposure and neurological degeneration were hypersensitive to killing by ultraviolet radiation [41,53].

### Cancer in XP patients

Our previous data have shown that 65% (69/106) of XP patients developed sunlight-induced skin cancers. Non-melanoma skin cancer was increased 10,000-fold and melanoma was increased 2,000-fold in patients under age 20 [1,2]. The XP-A, and XP-C patients developed hundreds of skin cancers (Table 1). The XP-A patient and XP-C patient XP24BE were treated with oral 13-cis-retinoic acid for chemoprevention of skin cancer [36]. They both had a good response, with no further skin malignancies identified at autopsy. The XP-D patient was sun protected from early childhood and developed only 9 skin cancers.

The XP-C patients developed internal cancers with a glioma in XP24BE and a cervical adenocarcinoma in XP1BE. XP patients have been estimated to have an 11-fold increase in internal neoplasms and a 50-fold increase in cancers of the central nervous system [2]. The reported tumors include Schwannoma in a 73 y/o [1], spinal cord astrocytoma in a 24 y/o, gastric cancer in a 50 y/o and lung cancer in a 37 y/o smoker [42]. Some carcinogenic components of cigarette smoke bind to DNA forming adducts that are repaired by the NER system. It is thought that the lung cancers in the young XP patients are related to mutations resulting from this unrepaired damage. The underlying mechanism of induction of the CNS neoplasms is not known.

### DNA repair defects in XP patients

Defects in the repair-enzyme genes *XPA* through *XPG* are known to cause XP [12,13,54]. Cells from XP patients with mutations in the *XPC* DNA repair gene have defective global genome DNA repair and normal transcription coupled repair. In contrast, cells from patients with mutations in the *CSA* or *CSB* genes have defects only in transcription coupled repair. These patients have clinical Cockayne syndrome with severe progressive neurological degeneration with short stature and retinal degeneration but without an increase in cancer [12,54]. However, cells from XP patients with mutations in the *XPA* and *XPD* genes have defective global genome repair and in addition, defective transcription coupled DNA repair. This additional transcription coupled DNA repair defect is associated with progressive neurological degeneration in humans (see for example [5]) and in mouse model systems [55]. Patients with trichothiodystrophy have mutations in the *XPD* gene in association with developmental abnormalities (including absent myelin in the brain) as well as sulfur deficient brittle hair, cataracts, bone abnormalities and increased susceptibility to infections, but no increase in cancer [12,54,56]. The level of DNA repair does not correlate with the clinical phenotype. Thus, while cells from XP-A patients with severe neurological degeneration generally have <2% of normal post-UV unscheduled DNA synthesis (UDS) in cultured skin fibroblasts, cells from XP-D patients, who also have severe neurological degeneration, have 25-50% UDS and cells from Cockayne syndrome patients have normal UDS. Further, cells from XP-C patients with no neurological abnormalities have 10% UDS [4,35,39,41,56]. Thus the relationship of mutations in the DNA repair genes to clinical phenotypes is complex and the precise mechanisms of these differences are not understood.

XP patients have defective repair of UV induced DNA damage to skin and eyes. Unrepaired DNA damage can lead to increased cell death and to a high frequency of somatic mutations in the surviving, dividing cells. This process is believed to underlie the skin sun sensitivity and high frequency of sunlight-induced cancers in XP patients. Skin tumors from XP patients frequently have evidence of UV type mutations [57,58]. There is a good correlation between post-UV killing and neurological degeneration in cultured cells from XP patients. Thus, skin fibroblasts from XP-A and XP-D patients with severe neurological degeneration are more sensitive to post-UV killing than are cells from XP-C patients who have no neurological abnormalities [41]. However, UV radiation does not reach the brain because of shielding from the scalp and bones of the skull. It has been hypothesized that oxidative DNA damage generated through metabolism or other sources might produce DNA damage that is not repaired in the non-dividing cells of the nervous system leading to progressive neuronal death [8]. Most types of oxidative DNA damage are repaired by base excision repair, a process that is not defective in XP cells. Indeed, both XP-C patients received therapeutic doses of x-radiation (which produces oxidative damage) for treatment (brain tumor in XP24BE and orbital tumors in XP1BE and spinal cord astrocytoma in another XP-C patient [42]) (Table 1) with a normal response. However, certain types of products of oxidative DNA damage, such as cyclopurine deoxynucleosides, are repaired by the NER system. Impaired repair of this type of oxidative DNA damage has been proposed as a mechanism of the progressive neurodegeneration seen in these XP patients [59,60].

## Conclusions

In conclusion, while all 4 XP patients had defects in NER genes, they had markedly different phenotypes with different clinical consequences. Three of the patients had hundreds of skin cancers early in life, and oral therapy with retinoids and increased sun protection proved effective in 2 of them. None of these XP patients died from their skin cancers. Two died from internal neoplasms (glioblastoma and endocervical adenocarcinoma) and the other two adult patients died from progressive neurodegeneration. Understanding the complex relationships of these DNA repair defects to these causes of death are a challenge for future investigations.

## Methods

### Neuropathological examination

The patients were examined at the NIH under protocols approved by the Institutional Review Board of the National Cancer Institute. They gave their permission for use of identifying images. The families of the patients have given their consent for performance of the autopsies. The brain, peripheral nerves, muscles were fixed with 10% buffered formalin and embedded in paraffin. Histological examinations were performed on 5 μm thick sections using hematoxylin–eosin (HE). Selected sections were evaluated with special stains (luxol fast blue (LFB), Bielschowsky, Masson’s trichrome) and immunostains (glial fibrillary acidic protein (GFAP), synaptophysin, NeuN, neurofilament proteins, KP-1, beta-amyloid, Isocitrate dehydrogenase 1 (IDH1), p53, EGFR, MIB-1). For immunohistochemical analyses, 5 μm thick sections were serially cut in selected brain regions and brain tumors. The control subjects had no neurological abnormalities and showed no morphological changes in the brain. Slides were incubated in Tris with 3% goat serum for 15 min and then incubated for 1–2 h at room temperature with primary antibody. Detection was carried out using an automated slide stainer (DAKO; Autostainer) with a horseradish peroxidase/3,3^′^-diaminobenzidine polymer-based detection system (DAKO; Envision+). No staining was observed in the sections incubated in the absence of either antibody.

## Abbreviations

BCC: Basal cell carcinomas; NER: Nucleotide excision repair; NIH: National Institutes of Health; SCC: squamous cell carcinoma; UDS: unscheduled DNA synthesis; UV: ultraviolet radiation; XP: xeroderma pigmentosum

## Competing interests

The authors declare no competing interests.

## Authors’ contributions

The autopsies were performed by J-PL, Y-CL, QL, PPA, KM, SH, DLL, DEK and JCO; The slides and images were interpreted by MMQ, MA, C-HL, MT, EJR, and JCO; The radiological images were interpreted by NP; The patients were cared for and clinical data collected by DT, JJD, HAF and KHK; DNA repair and mutations were determined by SK; The study was conceived and designed by MMQ, JCO, JJD and KHK. The manuscript was drafted and revised critically for intellectual content by J-PL, Y-CL, MMQ, EJR and KHK. All authors read and approved the final manuscript.
